# 2-Cyano-1-methyl­pyridinium nitrate

**DOI:** 10.1107/S1600536812019460

**Published:** 2012-05-05

**Authors:** Lynn V. Koplitz, Joel T. Mague, Michael N. Kammer, Cameron A. McCormick, Heather E. Renfro, David J. Vumbaco

**Affiliations:** a Department of Chemistry, Loyola University, New Orleans, LA 70118, USA; b Department of Chemistry, Tulane University, New Orleans, LA 70118, USA; c Department of Physics, Loyola University, New Orleans, LA 70118, USA; d Department of Biological Sciences, Loyola University, New Orleans, LA 70118, USA

## Abstract

In the title compound, C_7_H_7_N_2_^+^·NO_3_^−^, all atoms except the methyl H atoms lie on a crystallographic mirror plane. The inter­layer distance, including that between aligned N atoms from alternating cations and anions in adjacent layers, is exceptionally short at 3.055 (1) Å. Two-dimensional C—H⋯O hydrogen-bonded networks link cations to anions, while C—H⋯N inter­actions link cations within each layer. Anion–π inter­actions with the cations assist in binding the layers together.

## Related literature

For the structure of 2-cyano­anilinium nitrate, see: Cui & Wen (2008 ▶). For the structures of other 2- and 3-cyano­anilinium salts, see: Zhang (2009 ▶); Wang (2009*a* ▶,*b* ▶); Wen (2008 ▶). For previous work on cyano-*N*-methyl­pyridinium salts, see: Koplitz *et al.* (2003 ▶); Mague *et al.* (2005 ▶). For a discussion of anion–π inter­actions, see: Frontera *et al.* (2011 ▶).
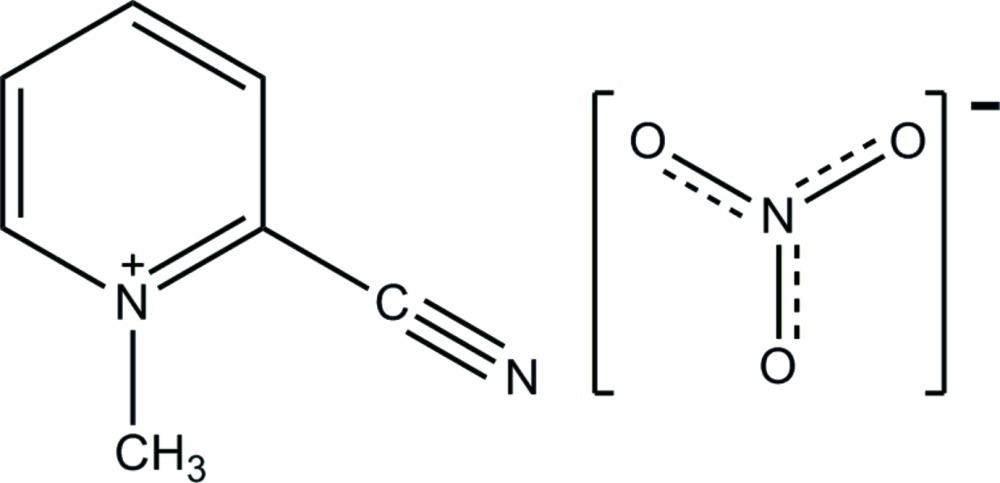


## Experimental

### 

#### Crystal data

C_7_H_7_N_2_^+^·NO_3_^−^
*M* *_r_* = 181.16
Orthorhombic, 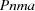

*a* = 16.302 (3) Å
*b* = 6.1012 (10) Å
*c* = 8.0318 (13) Å
*V* = 798.9 (2) Å^3^
*Z* = 4
Mo *K*α radiation
μ = 0.12 mm^−1^
*T* = 160 K
0.22 × 0.14 × 0.13 mm

#### Data collection

Bruker SMART APEX CCD diffractometer
Absorption correction: multi-scan (*TWINABS*; Sheldrick, 2009 ▶) *T*_min_ = 0.652, *T*_max_ = 0.985
25942 measured reflections
1143 independent reflections
1005 reflections with *I* > 2σ(*I*)
*R*_int_ = 0.069

#### Refinement

*R*[*F* ^2^ > 2σ(*F* ^2^)] = 0.059
*wR*(*F* ^2^) = 0.156
*S* = 1.14
1143 reflections
80 parameters
H-atom parameters constrained
Δρ_max_ = 0.46 e Å^−3^
Δρ_min_ = −0.78 e Å^−3^

### 

Data collection: *APEX2* (Bruker, 2010 ▶); cell refinement: *SAINT* (Bruker, 2009 ▶); data reduction: *SAINT* and *CELL_NOW* (Sheldrick, 2008*b* ▶); program(s) used to solve structure: *SHELXS97* (Sheldrick, 2008*a* ▶); program(s) used to refine structure: *SHELXL97* (Sheldrick, 2008*a* ▶); molecular graphics: *SHELXTL* (Sheldrick, 2008*a* ▶); software used to prepare material for publication: *SHELXTL*.

## Supplementary Material

Crystal structure: contains datablock(s) I, global. DOI: 10.1107/S1600536812019460/hb6770sup1.cif

Structure factors: contains datablock(s) I. DOI: 10.1107/S1600536812019460/hb6770Isup2.hkl

Supplementary material file. DOI: 10.1107/S1600536812019460/hb6770Isup3.cml

Additional supplementary materials:  crystallographic information; 3D view; checkCIF report

## Figures and Tables

**Table 1:** Hydrogen-bond geometry (Å, °)

*D*—H⋯*A*	*D*—H	H⋯*A*	*D*⋯*A*	*D*—H⋯*A*
C2—H2⋯O1	0.95	2.34	3.227 (2)	155
C3—H3⋯O2	0.95	2.48	3.276 (2)	141
C4—H4⋯O1^i^	0.95	2.37	3.215 (2)	148
C7—H7*B*⋯O3^ii^	0.98	2.38	3.247 (2)	148
C7—H7*A*⋯O2^iii^	0.98	2.67	3.326 (2)	125
C7—H7*C*⋯O2^iv^	0.98	2.64	3.3503 (11)	130
C1—H1⋯N2^v^	0.95	2.67	3.259 (2)	123
C2—H2⋯N2^v^	0.95	2.62	3.283 (2)	125

Symmetry codes: (i) 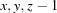; (ii) 
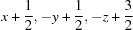
; (iii) 
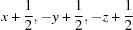
; (iv) 
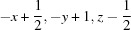
; (v) 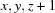.

**Fig. 1.**
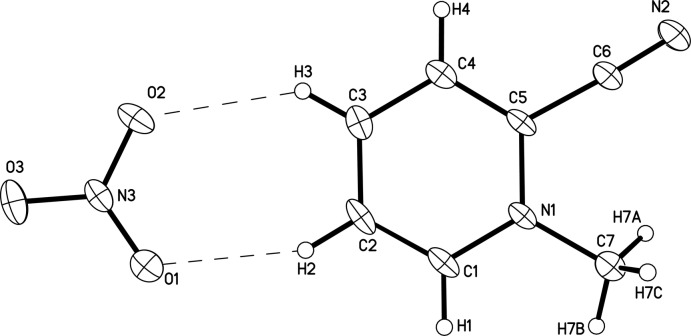
Perspective view of the asymmetric unit (50% probability ellipsoids) showing the intralayer hydrogen bonding.

**Fig. 2.**
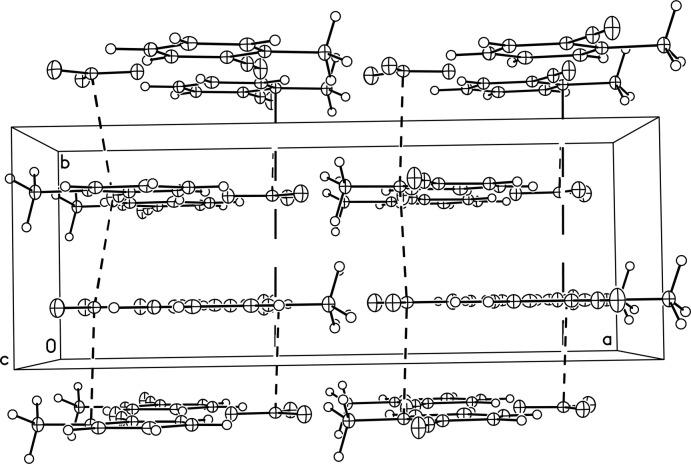
The packing viewed down the c axis showing the interlayer interactions.

## supplementary crystallographic information

### Comment

Both the chloride and bromide salts of the 3-cyano-*N*-methylpyridinium cation possess crystallographic mirror symmetry with all atoms except for the methyl H atoms lying in the mirror planes (Koplitz *et al.*, 2003; Mague *et al.*, 2005). More recently, Cui and Wen reported that 2-cyanoanilinium nitrate also crystallizes in flat layers of two-dimensional networks with only a few atoms, including nitrate O atoms and ammonium H atoms, protruding from the mirror planes (Cui & Wen, 2008). The similarities between cations suggested a systematic study of analogous cyanopyridinium and anilinium salts both to look for other layered structures resembling graphite and to investigate trends in crystal architecture with variations in anion as well as relative ring position of the cyano and pendant groups. Of the eight possibilities investigated so far, the title compound is the only additional layered structure discovered to date.

2-Cyano-*N*-methylpyridinium nitrate crystallizes in the same space group as 2-cyanoanilinium nitrate. However, even though the cations of these two compounds are isomers that differ only by the interchange of one carbon and one nitrogen atom from the ring and pendant group, the distribution of anions relative to cations in the crystal differs markedly. In particular, the nitrate ions in the present structure lie wholly in the mirror plane such that the N3—O1 bond of one anion is oriented with O1 lying directly over the centroid of the pyridinium ring in the adjacent layer and N3 lying directly over the pyridinium nitrogen (N1) at a distance of 3.055 (1) Å. This close contact is likely the result of electrostatic cation-anion attraction with the orientation reinforced by an anion-π interaction (Frontera *et al.*, 2011). Calculated densities are 1.401 g-cm^-3^ for 2-cyanoanilinium nitrate and 1.531 g-cm^-3^ for 2-cyano-*N*-methylpyridinium nitrate with the greater density of the latter attributable to the anion lying wholly in the mirror plane rather than perpendicular to it. This is reflected in the much shorter *b* axis of the unit cell [6.101 (1) *versus*. 6.563 (1) Å] which is the stacking direction in the pyridinium salt. In the anilinium salt, only one nitrate N—O bond is coplanar with the cation rings while the other two oxygen atoms are disposed on either side of the mirror and form N—H···O hydrogen bonds with the pendant ammonium groups of the cations in adjacent layers. These strong hydrogen bonds are likely responsible for the different orientation of the anion in the anilinium salt which leads to a larger interlayer spacing.

Interlayer distances (in Å) for comparison: graphite, 3.35; 3-cyano-*N*-methylpyridinium bromide, 3.313 (4); 2-cyanoanilinium nitrate, 3.281 (2); 3-cyano-*N*-methylpyridinium chloride, 3.201 (4); 2-cyano-*N*-methylpyridinium nitrate, 3.055 (1).

### Experimental

2-Cyanopyridine (10.5 g) was first melted in a warm water bath and then dissolved in benzene (40 ml). Iodomethane (9.5 ml) was added to this solution slowly with stirring and the solution was refluxed for 2 h. Yellow solid 2-cyano-*N*-methyl pyridinium iodide (m.p. 146–150° C) was collected by vacuum filtration. This solid was then reacted with an equimolar amount of AgNO_3_ in ethanol and the AgI precipitate removed by vacuum filtration. The filtrate containing 2-cyano-*N*-methyl nitrate was slowly evaporated to dryness to form colourless blocks of the title compound.

### Refinement

H-atoms were placed in calculated positions (C—H = 0.95 - 0.98 Å) and included as riding contributions with isotropic displacement parameters 1.2 - 1.5 times those of the attached carbon atoms. Because both ions sit on the mirror plane, the methyl group H atoms are disordered across the mirror. Trial refinements with both the one-component reflection file extracted from the full data set with TWINABS and with the full two-component file showed that use of the former provided a better refinement.

### Crystal data

**Table:**

C_7_H_7_N_2_^+^·NO_3_^−^	*F*(000) = 376
*M**_r_* = 181.16	*D*_x_ = 1.506 Mg m^−^^3^
Orthorhombic, *P**n**m**a*	Mo *K*α radiation, λ = 0.71073 Å
Hall symbol: -P 2ac 2n	Cell parameters from 1462 reflections
*a* = 16.302 (3) Å	θ = 2.5–28.1°
*b* = 6.1012 (10) Å	µ = 0.12 mm^−^^1^
*c* = 8.0318 (13) Å	*T* = 160 K
*V* = 798.9 (2) Å^3^	Block, colourless
*Z* = 4	0.22 × 0.14 × 0.13 mm

### Data collection

**Table:**

Bruker SMART APEX CCD [or Bruker APEXII CCD?]diffractometer	1143 independent reflections
Radiation source: fine-focus sealed tube	1005 reflections with *I* > 2σ(*I*)
Graphite monochromator	*R*_int_ = 0.069
φ and ω scans	θ_max_ = 29.1°, θ_min_ = 2.5°
Absorption correction: multi-scan (TWINABS; Sheldrick, 2009)	*h* = 0→22
*T*_min_ = 0.652, *T*_max_ = 0.985	*k* = 0→8
25942 measured reflections	*l* = 0→10

### Refinement

**Table:**

Refinement on *F*^2^	Primary atom site location: structure-invariant direct methods
Least-squares matrix: full	Secondary atom site location: difference Fourier map
*R*[*F*^2^ > 2σ(*F*^2^)] = 0.059	Hydrogen site location: inferred from neighbouring sites
*wR*(*F*^2^) = 0.156	H-atom parameters constrained
*S* = 1.14	*w* = 1/[σ^2^(*F*_o_^2^) + (0.0984*P*)^2^ + 0.1829*P*] where *P* = (*F*_o_^2^ + 2*F*_c_^2^)/3
1143 reflections	(Δ/σ)_max_ < 0.001
80 parameters	Δρ_max_ = 0.46 e Å^−^^3^
0 restraints	Δρ_min_ = −0.78 e Å^−^^3^

### Special details

**Table:**

Experimental. The diffraction data were obtained from 3 sets of 400 frames, each of width 0.5 °. in omega, colllected at phi = 0.00, 90.00 and 180.00 °. and 2 sets of 800 frames, each of width 0.45 ° in phi, collected at omega = -30.00 and 210.00 °. The scan time was sec/frame. Analysis of 576 reflections having I/σ(I) > 15 and chosen from the full data set with *CELL_NOW* (Sheldrick, 2008*b*) showed the crystal to belong to the orthorhombic system and to be twinned by a 180 ° rotation about *c*. The raw data were processed using the multi-component version of *SAINT* under control of the two-component orientation file generated by *CELL_NOW*.
Geometry. All e.s.d.'s (except the e.s.d. in the dihedral angle between two l.s. planes) are estimated using the full covariance matrix. The cell e.s.d.'s are taken into account individually in the estimation of e.s.d.'s in distances, angles and torsion angles; correlations between e.s.d.'s in cell parameters are only used when they are defined by crystal symmetry. An approximate (isotropic) treatment of cell e.s.d.'s is used for estimating e.s.d.'s involving l.s. planes.
Refinement. Refinement of *F*^2^ against ALL reflections. The weighted *R*-factor *wR* and goodness of fit *S* are based on *F*^2^, conventional *R*-factors *R* are based on *F*, with *F* set to zero for negative *F*^2^. The threshold expression of *F*^2^ > σ(*F*^2^) is used only for calculating *R*-factors(gt) *etc*. and is not relevant to the choice of reflections for refinement. *R*-factors based on *F*^2^ are statistically about twice as large as those based on *F*, and *R*- factors based on ALL data will be even larger.

### Fractional atomic coordinates and isotropic or equivalent isotropic displacement parameters (Å^2^)

**Table:**

	*x*	*y*	*z*	*U*_iso_*/*U*_eq_	Occ. (<1)
N1	0.39751 (10)	0.2500	0.27067 (17)	0.0183 (4)	
N2	0.38257 (11)	0.2500	−0.1590 (2)	0.0370 (5)	
C1	0.37563 (12)	0.2500	0.4325 (2)	0.0217 (4)	
H1	0.4168	0.2500	0.5163	0.026*	
C2	0.29366 (12)	0.2500	0.4776 (2)	0.0241 (4)	
H2	0.2788	0.2500	0.5920	0.029*	
C3	0.23350 (12)	0.2500	0.3566 (2)	0.0226 (4)	
H3	0.1772	0.2500	0.3870	0.027*	
C4	0.25634 (11)	0.2500	0.1886 (2)	0.0214 (4)	
H4	0.2160	0.2500	0.1033	0.026*	
C5	0.33831 (11)	0.2500	0.1499 (2)	0.0192 (4)	
C6	0.36487 (12)	0.2500	−0.0215 (2)	0.0242 (4)	
C7	0.48583 (12)	0.2500	0.2249 (2)	0.0249 (4)	
H7A	0.4945	0.1532	0.1292	0.037*	0.50
H7B	0.5184	0.1975	0.3194	0.037*	0.50
H7C	0.5029	0.3993	0.1961	0.037*	0.50
N3	0.11091 (10)	0.2500	0.78367 (18)	0.0212 (4)	
O1	0.18594 (9)	0.2500	0.81475 (19)	0.0292 (4)	
O2	0.08669 (10)	0.2500	0.63510 (17)	0.0329 (4)	
O3	0.06020 (10)	0.2500	0.9001 (2)	0.0383 (4)	

### Atomic displacement parameters (Å^2^)

**Table:**

	*U*^11^	*U*^22^	*U*^33^	*U*^12^	*U*^13^	*U*^23^
N1	0.0241 (7)	0.0219 (7)	0.0090 (7)	0.000	−0.0007 (5)	0.000
N2	0.0305 (10)	0.0696 (14)	0.0110 (8)	0.000	−0.0001 (7)	0.000
C1	0.0325 (9)	0.0237 (8)	0.0090 (8)	0.000	−0.0007 (7)	0.000
C2	0.0378 (11)	0.0254 (9)	0.0092 (8)	0.000	0.0048 (7)	0.000
C3	0.0260 (9)	0.0262 (8)	0.0154 (8)	0.000	0.0050 (6)	0.000
C4	0.0261 (9)	0.0253 (9)	0.0127 (8)	0.000	−0.0017 (6)	0.000
C5	0.0263 (9)	0.0232 (8)	0.0081 (8)	0.000	−0.0019 (6)	0.000
C6	0.0241 (8)	0.0368 (10)	0.0118 (8)	0.000	−0.0005 (6)	0.000
C7	0.0233 (9)	0.0353 (10)	0.0161 (9)	0.000	−0.0005 (6)	0.000
N3	0.0283 (8)	0.0231 (7)	0.0123 (7)	0.000	0.0030 (5)	0.000
O1	0.0271 (8)	0.0407 (8)	0.0198 (7)	0.000	−0.0006 (5)	0.000
O2	0.0369 (9)	0.0490 (9)	0.0128 (7)	0.000	−0.0044 (5)	0.000
O3	0.0383 (9)	0.0558 (10)	0.0207 (8)	0.000	0.0152 (6)	0.000

### Geometric parameters (Å, º)

**Table:**

N1—C1	1.348 (2)	C4—C5	1.372 (3)
N1—C5	1.368 (2)	C4—H4	0.9500
N1—C7	1.486 (2)	C5—C6	1.443 (2)
N2—C6	1.142 (2)	C7—H7A	0.9800
C1—C2	1.384 (3)	C7—H7B	0.9800
C1—H1	0.9500	C7—H7C	0.9800
C2—C3	1.381 (3)	N3—O3	1.248 (2)
C2—H2	0.9500	N3—O1	1.248 (2)
C3—C4	1.399 (2)	N3—O2	1.257 (2)
C3—H3	0.9500		
			
C1—N1—C5	119.79 (16)	C3—C4—H4	120.7
C1—N1—C7	119.66 (15)	N1—C5—C4	121.77 (17)
C5—N1—C7	120.55 (15)	N1—C5—C6	117.69 (16)
N1—C1—C2	120.52 (17)	C4—C5—C6	120.55 (16)
N1—C1—H1	119.7	N2—C6—C5	177.2 (2)
C2—C1—H1	119.7	N1—C7—H7A	109.5
C3—C2—C1	120.08 (16)	N1—C7—H7B	109.5
C3—C2—H2	120.0	H7A—C7—H7B	109.5
C1—C2—H2	120.0	N1—C7—H7C	109.5
C2—C3—C4	119.32 (18)	H7A—C7—H7C	109.5
C2—C3—H3	120.3	H7B—C7—H7C	109.5
C4—C3—H3	120.3	O3—N3—O1	119.95 (16)
C5—C4—C3	118.52 (17)	O3—N3—O2	120.21 (17)
C5—C4—H4	120.7	O1—N3—O2	119.84 (16)

### Hydrogen-bond geometry (Å, º)

**Table:**

*D*—H···*A*	*D*—H	H···*A*	*D*···*A*	*D*—H···*A*
C2—H2···O1	0.95	2.34	3.227 (2)	155
C3—H3···O2	0.95	2.48	3.276 (2)	141
C4—H4···O1^i^	0.95	2.37	3.215 (2)	148
C7—H7*B*···O3^ii^	0.98	2.38	3.247 (2)	148
C7—H7*A*···O2^iii^	0.98	2.67	3.326 (2)	125
C7—H7*C*···O2^iv^	0.98	2.64	3.3503 (11)	130
C1—H1···N2^v^	0.95	2.67	3.259 (2)	123
C2—H2···N2^v^	0.95	2.62	3.283 (2)	125

Symmetry codes: (i) *x*, *y*, *z*−1; (ii) *x*+1/2, −*y*+1/2, −*z*+3/2; (iii) *x*+1/2, −*y*+1/2, −*z*+1/2; (iv) −*x*+1/2, −*y*+1, *z*−1/2; (v) *x*, *y*, *z*+1.
